# Targeting Bruton’s Tyrosine Kinase in CLL: Selectivity, Resistance Mechanisms, and Emerging Therapeutic Strategies

**DOI:** 10.3390/cells15151383

**Published:** 2026-07-31

**Authors:** Eduardo Bravo, Claudia Cabrera Pastrana, Erica N. Lamkin, Katarzyna Ciurko, Justin Taylor

**Affiliations:** 1 Sylvester Comprehensive Cancer Center, University of Miami Miller School of Medicine, Miami, FL 33136, USA; exb1333@med.miami.edu (E.B.J.); cxc534@med.miami.edu (C.C.P.); enl55@med.miami.edu (E.N.L.); kxc2961@med.miami.edu (K.C.); 2 Medical Scientist Training Program, University of Miami Miller School of Medicine, Miami, FL 33136, USA; 3 Sheila and David Fuente Graduate Program in Cancer Biology, University of Miami Miller School of Medicine, Miami, FL 33136, USA

**Keywords:** Bruton’s tyrosine kinase, BTK, inhibitor, degrader, proteolysis-targeting chimera, PROTAC, chronic lymphocytic leukemia, CLL, B-cell receptor, B-cell malignancy

## Abstract

Kinases are highly explored drug targets due to their central role in cell growth, differentiation, and cell death, as well as their relationship to cancer initiation and progression. However, with more than 500 kinases in the human kinome and significant structural similarities among them, kinases present a major selectivity challenge. Bruton’s tyrosine kinase (BTK), a highly studied kinase, plays an important role in the B-cell receptor pathway. Overexpression of BTK in B cells has been linked to the development of B-cell lymphomas, like chronic lymphocytic leukemia (CLL), as well as certain autoimmune diseases. For this reason, BTK is an attractive therapeutic target. In this review, we will provide a summary of the design of irreversible inhibitors, reversible inhibitors, and Proteolysis-targeting chimera (PROTAC) degraders targeting BTK. We will include crystal structures of compounds bound to BTK^WT^ and provide a review of how the design of these inhibitors and PROTACs leads to a more selective inhibition and degradation of BTK. With the emergence of identified BTK resistance mutations, alternative strategies beyond established BTK inhibitors are fundamental for designing more selective inhibitors and degraders that can overcome resistance.

## 1. Introduction

As key components of cellular signaling pathways, kinases regulate numerous biological processes, and perturbations in their activity can contribute to oncogenic signaling and tumor development [1,2,3,4]. With the approval of the 100th kinase inhibitor [5,6,7], this highlights the importance of kinases as therapeutic targets in drug discovery. However, with over 518 kinases in the human kinome, many of which share highly conserved structural features and participate in interconnected signaling networks, achieving selectivity remains a major challenge in kinase drug discovery [8,9,10,11,12,13,14,15,16,17]. Bruton’s tyrosine kinase is one of the most extensively studied targets in drug discovery programs. BTK is a cytoplasmic, non-receptor tyrosine kinase that plays a critical role in B-cell development, differentiation, and signaling. It belongs to the TEC family of kinases comprising BTK, BMX, ITK, TEC, and TXK [18,19,20,21,22,23,24,25]. The biological significance of BTK is largely attributed to its central role in B-cell receptor (BCR) signaling, where it functions as a critical mediator linking antigen recognition to downstream pathways.

**Figure 1:**
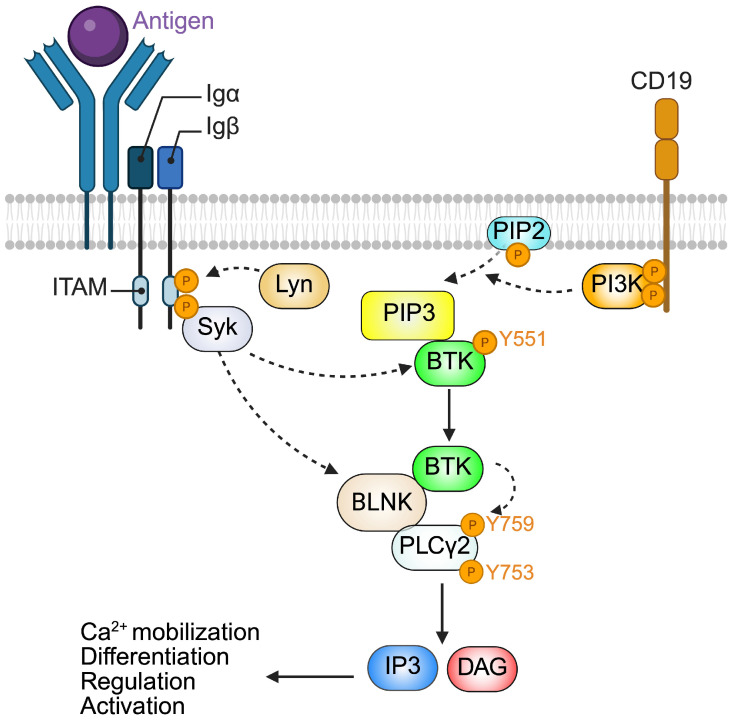
B-cell receptor pathway. Activated BTK phosphorylates PLCγ2, leading to calcium mobilization and signaling pathways that regulate B-cell differentiation, regulation, and activation. Dysregulation of this pathway contributes to the development of B-cell malignancies and autoimmune diseases [27]. Created in BioRender. Bravo Jr., E. (2026) https://BioRender.com/ojd02jw (accessed on 27 July 2026).

Following antigen binding to the BCR membrane receptor, inside the cell, the Src family kinase Lyn initiates signaling by phosphorylating the immunoreceptor tyrosine-based activation motif (ITAM) of the BCR as well as CD19 (Figure 1). The phosphorylation of these two sites serves as docking sites to phosphorylate additional kinases such as phosphatidylinositol-3 kinase (PI3K) and spleen tyrosine kinase (Syk). Activated PI3K phosphorylates phosphatidylinositol-4,5-biphosphate (PIP2) into phosphatidylinositol-3,4,5-triphosphate (PIP3), a critical step since PIP3 recruits BTK to the cell membrane and promotes BTK phosphorylation at Tyr551, which induces autophosphorylation at Tyr223. Also, Syk phosphorylates BLNK, which functions as a scaffold to bring together activated BTK to phosphorylate PLC_ϒ_2, leading to a cascade of signaling events involving Ca^2+^ mobilization, differentiation, regulation, and activation [3,26,27].

Given its central role in B-cell signaling, dysregulation of BTK has been implicated in the development of B-cell malignancies, autoimmune diseases and primary immunodeficiencies, most importantly, x-linked agammaglobulinemia [20,24,28,29,30,31]. Consequently, significant drug discovery efforts have focused on targeting BTK, particularly through covalent inhibition of the C481 residue located within the ATP-binding pocket [24,32,33,34,35,36]. This strategy led to the development of first-generation irreversible inhibitors such as Ibrutinib [37]. However, given that cysteine is present in other kinases (BMX, ITK, TEC, BLK, EGFR, ERBB2, and JAK3), achieving selectivity was a challenge [32,38,39,40,41,42]. This broad kinase inhibition profile is the primary driver of its distinctive toxicity pattern. Specifically, Ibrutinib is associated with elevated rates of atrial fibrillation [43], hypertension [44], and bleeding [45]. Mechanistically, ibrutinib-induced atrial fibrillation is multifactorial: off-target inhibition of CSK leads to dysregulated Src family kinase activity and atrial remodeling, while inhibition of TEC and BTK itself downregulates the PI3K-Akt cardioprotective pathway [43,46], and enhanced CaMKII [43,47,48] activity promotes calcium dysregulation and triggered arrhythmia. Hypertension is attributed to the off-target inhibition of ERBB2, BLK, and SRC through oxidative stress and endothelial dysfunction pathways [49]. Notably, patients with X-linked agammaglobulinemia who completely lack BTK do not exhibit a bleeding diathesis, yet BTK/TEC [45,50,51] double-knockout mice show complete loss of collagen-induced platelet aggregation, strongly suggesting that ibrutinib’s bleeding complications arise from the simultaneous inhibition of both BTK and TEC in platelets rather than from BTK inhibition alone.

To address this limitation, second-generation irreversible inhibitors such as Acalabrutinib and Zanubrutinib were developed with improved kinase selectivity profiles [52,53]. In head-to-head phase III trials (ALPINE, ELEVATE-RR), second-generation agents demonstrated significantly lower rates [54,55,56], of atrial fibrillation, bleeding, and treatment discontinuation compared with ibrutinib, without compromising efficacy. These findings validate the medicinal chemistry strategy of optimizing selectivity within the ATP-binding site: structural modifications that reduced off-target activity translate directly into improved clinical tolerability. Despite their clinical success, the emergence of resistance mutations, most notably C481S, substantially reduces the efficacy of irreversible BTK inhibitors because covalent bond formation with C481 is required for irreversible target engagement. This challenge led to the development of reversible, non-covalent BTK inhibitors, including GDC-0853 (Fenebrutinib) [57], GDC-0834 [58], BMS-986142 [59], BMS-935177 [60], and Pirtobrutinib [61]. While these inhibitors overcome C481S resistance, additional mutations have subsequently emerged that disrupt critical inhibitor–protein interactions and thus prevent inhibitor binding [62].

More recently, studies have revealed that BTK possesses kinase-dead functions that contribute to continuous B-cell signaling [63,64]. In these contexts, catalytic inhibition alone may be insufficient because BTK can continue to support signaling through protein–protein interactions mediated by other structural regions or domains. These findings have further stimulated interest in targeted protein degradation as a therapeutic strategy. Proteolysis-targeting chimeras (PROTACs) offer the potential to eliminate both the catalytic and scaffolding functions of BTK by inducing complete protein degradation [2,62,65,66,67,68,69,70]. Examples of this approach include degraders such as NX-2127 and other next-generation BTK-targeting degraders currently under investigation [63,71,72].

In this review, we highlight the key molecular interactions that govern the binding of BTK inhibitors and degraders, with particular emphasis on how the engagement of distinct binding pockets influences potency, selectivity, and resistance profiles. By examining the structural basis of inhibitor recognition and resistance, we aim to provide a framework for addressing current resistance-associated mutations.

## 2. Irreversible BTK Selectivity

**Figure 2:**
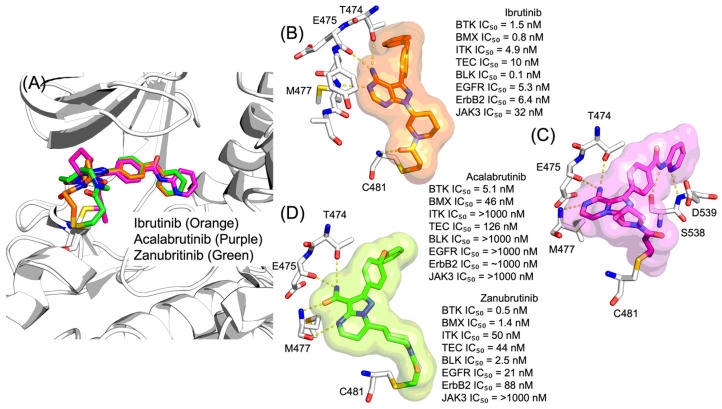
Selectivity of irreversible inhibitors against cysteine-containing kinases (**A**). (**B**) First-(Orange, Ibrutinib, PDB: 5P9J, [76]) and (**C**) second-generation (Purple, Acalabrutinib, PDB: 8FD9 [77]; (**D**) Green, Zanubrutinib, PDB: 6J6M [78].) irreversible BTK inhibitors target C481 within the ATP-binding site of BTK and related kinases that contain a homologous cysteine residue. Although these inhibitors share a common irreversible binding mechanism, they exhibit distinct kinase selectivity profiles. Enhanced selectivity minimizes off-target kinase inhibition while maintaining potent suppression of B-cell receptor signaling, thereby improving therapeutic efficacy and safety. This figure was made with Pymol (PyMOL. The PyMOL Molecular Graphics System, Version 2.5 Schrödinger, LLC, 2015).

The first-in-class BTK inhibitor, Ibrutinib (BTK IC_50_ = 1.5 nM, Figure 2B), was approved by the FDA in 2013 for the treatment of mantle cell lymphoma (MCL), and in 2014 for chronic lymphocytic leukemia (CLL). Its binding mode within BTK is characterized by three hydrogen-bond interactions in the ATP-binding site involving T474, E475, and M477. In the solvent-exposed region, it forms a hydrogen bond with the backbone NH of C481 and subsequently forms an irreversible covalent bond with the thiol side chain of C481. Although ibrutinib exhibits potent inhibition of BTK, it also inhibits several other cysteine-containing kinases, including BMX (IC_50_ = 0.8 nM), ITK (IC_50_ = 4.9 nM), TEC (IC_50_ = 10 nM), BLK (IC_50_ = 0.1 nM), EGFR (IC_50_ = 5.3 nM), ErbB2 (IC_50_ = 6.4 nM), and JAK3 (IC_50_ = 32 nM) [37,73,74,75].

Acalabrutinib (Figure 2C) and Zanubrutinib (Figure 2D) are second-generation BTK inhibitors designed to improve selectivity while maintaining potent BTK inhibition. Acalabrutinib (BTK IC_50_ = 5.1 nM) binds in a similar manner to ibrutinib but extends further into the back pocket, forming additional interactions with residues T474, E475, M477, S538, and D539. This expanded interaction network contributes to improved kinase selectivity, as reflected by its activity profile against kinases containing a cysteine residue analogous to BTK C481 (BMX IC_50_ = 46 nM, ITK IC_50_ > 1000 nM, TEC IC_50_ = 126 nM, BLK IC_50_ > 1000 nM, EGFR IC_50_ > 1000 nM, ErbB2 IC_50_ ≈ 1000 nM, and JAK3 IC_50_ > 1000 nM) [52,79,80,81].

Zanubrutinib (BTK IC_50_ = 0.5 nM) is structurally related to ibrutinib but adopts a distinct binding orientation within the hinge region. It forms four hydrogen-bond interactions involving T474, E475, and M477, resulting in enhanced selectivity compared with ibrutinib (BMX IC_50_ = 1.4 nM, ITK IC_50_ = 50 nM, TEC IC_50_ = 44 nM, BLK IC_50_ = 2.5 nM, EGFR IC_50_ = 21 nM, ErbB2 IC_50_ = 88 nM, and JAK3 IC_50_ > 1000 nM) [53,78].

The reported biochemical IC_50_ values for the irreversible BTK inhibitors discussed were determined using the same biochemical kinase assay based on the immobilized metal ion affinity-based fluorescence polarization (IMAP) platform under identical experimental conditions as described by Barf et al. [52]. Briefly, BTK enzyme was preincubated with inhibitors for 1 h at room temperature prior to initiating the reaction with ATP at its apparent K_m_ concentration (5 μM final) and a fluorescein-labeled peptide substrate. Enzymatic activity was measured after a 2 h reaction using fluorescence polarization, and values were calculated as the percentage of the difference in readout of the controls with and without ATP. IC_50_ values were determined by curve fitting of the experimental results. Collectively, these data demonstrate that although ibrutinib, acalabrutinib, and zanubrutinib all target the BTK hinge region through interactions with T474, E475, and M477, subtle differences in how they occupy the ATP-binding site significantly influence kinase selectivity. In particular, acalabrutinib extends further into the back pocket and explores a larger portion of the available chemical space, leading to substantially improved selectivity across the kinome while retaining potent BTK inhibition.

## 3. Reversible BTK Selectivity

Irreversible BTK inhibition proved highly effective until the emergence of the C481S mutation, which presented a new challenge for medicinal chemists. Most irreversible BTK inhibitors rely on covalent bond formation between an acrylamide warhead and the thiol side chain of C481 [82]. Therefore, the mutation of cysteine to serine reduces the effectiveness of covalent inhibitors and prompted medicinal chemistry efforts to identify alternative binding strategies within the BTK domain. In this context, reversible inhibitors emerged as an effective approach. Most reversible BTK inhibitors engage the hinge region and extend into additional regions of the kinase domain, such as the back pocket or H3 pocket, to improve potency and selectivity [42,83,84,85].

**Figure 3:**
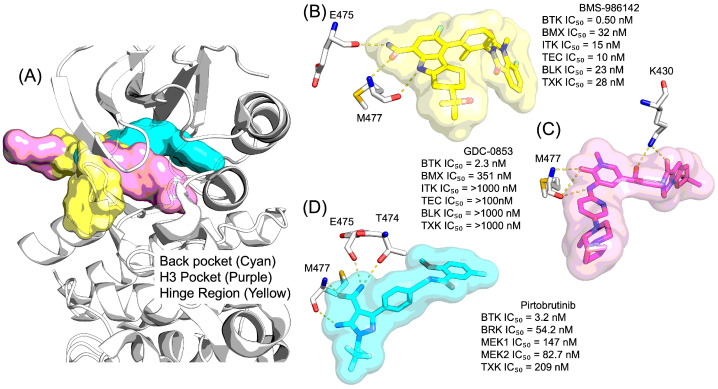
Aligned PDB structures of the BTK domain showing the distinct binding modes of reversible BTK inhibitors (**A**). Reversible inhibitors can engage (**B**) the hinge region (Yellow, BMS-986142, PDB: 5T18, [59]), (**C**) H3 pocket (Purple, GDC-0853, PDB: 5VFI, [57]), or (**D**) the back pocket (Cyan, Pirtobrutinib, PDB:8FLL, [61]) of BTK. Targeting the H3 pocket enhances kinase selectivity by exploiting non-conserved region within BTK, thereby reducing off-target activity against related kinases while maintaining potent inhibition. This figure was made with Pymol (PyMOL. The PyMOL Molecular Graphics System, Version 2.5 Schrödinger, LLC, 2015).

BMS-935177 and BMS-986142 (Figure 3B) are two reversible hinge-binding BTK inhibitors developed by Bristol Myers Squibb. BMS-935177 (BTK IC_50_ = 3 nM) forms two hydrogen bonds with M477: one through the carbonyl group of BMS-935177 and a second through the NH of the carbazole. A third hydrogen bond is formed between E475 and the NH of the amide group. In a selectivity assay, BMS-935177 inhibited BMX, ITK, TEC, BLK, and TXK with IC_50_ values of 24 nM, 96 nM, 13 nM, 20 nM, and 180 nM, respectively. BMS-986142 (BTK IC_50_ = 0.50 nM) is an optimized analog of BMS-935177 developed to improve tolerability, potency, and selectivity. It maintains similar hinge interactions with M477 and E475 but differs structurally through the incorporation of a fluorophenyl group and a quinazolinone ring, which improve its pharmacokinetic properties. Compared with BMS-935177, BMS-986142 inhibits BMX, ITK, TEC, BLK, and TXK with IC_50_ values of 32 nM, 15 nM, 10 nM, 23 nM, and 28 nM, respectively [59,60].

Because selectivity remains a major challenge in BTK inhibitor design, another strategy has focused on targeting the H3 pocket [86,87]. This pocket contains sequence and structural differences compared with other kinases, making it an attractive region for achieving improved BTK selectivity. GDC-0834 (BTK IC_50_ = 6 nM) forms three hydrogen bonds with M477 in the hinge region through its pyrazinone group. In the H3 pocket, its amide group forms a hydrogen bond with K430, while additional groups are stabilized through water-mediated hydrogen-bonding interactions. In a kinase selectivity panel, GDC-0834 showed only a three-fold selectivity difference over BMX; however, it displayed weaker selectivity against most other kinases. Because GDC-0834 underwent rapid amide-bond hydrolysis in humans, further optimization led to the discovery of GDC-0853 [58,88].

GDC-0853 (BTK IC_50_ = 2.3 nM, Figure 3C) preserves the hinge-binding interactions with M477 while improving H3 pocket engagement. Its hydroxymethyl group forms hydrogen bonds with K430, and its tricyclic ring system extends deeper into the H3 pocket. In selectivity screening, GDC-0853 inhibited BMX with an IC_50_ of 351 nM, while ITK, TEC, BLK, and TXK all showed IC_50_ values of approximately 1000 nM or greater, indicating improved selectivity compared with earlier reversible BTK inhibitors [57].

Pirtobrutinib (BTK IC_50_ = 3.2 nM, Figure 3D) binds the hinge region through hydrogen bonds with E475, T474 and M477 and extends into the back pocket toward the αC-helix. By extending to the back pocket, it enables additional water-mediated bonding to K430 and D539. In kinase selectivity screening, pirtobrutinib inhibited BRK, MEK1, MEK2, and TXK with IC_50_ values of 54.2 nM, 147 nM, 82.7 nM, and 209 nM, respectively [61].

Although the IC_50_ values for these inhibitors were measured under different assay conditions and across different kinase panels, the overall trend suggests that the targeting of BTK through the H3 pocket is a promising strategy for achieving selective reversible BTK inhibition. Clinically, pirtobrutinib demonstrated durable responses in patients with CLL previously treated with covalent BTK inhibitors, including those harboring C481S mutations, validating the reversible binding strategy [89].

## 4. Proteolysis Targeting Chimeras (PROTACs)

Both irreversible and reversible inhibition strategies have provided effective solutions to the challenges associated with targeting BTK. These medicinal chemistry approaches have led to the development of several successful therapeutics, including the FDA-approved reversible BTK inhibitor pirtobrutinib. However, BTK continues to present new challenges for drug discovery.

**Figure 4:**
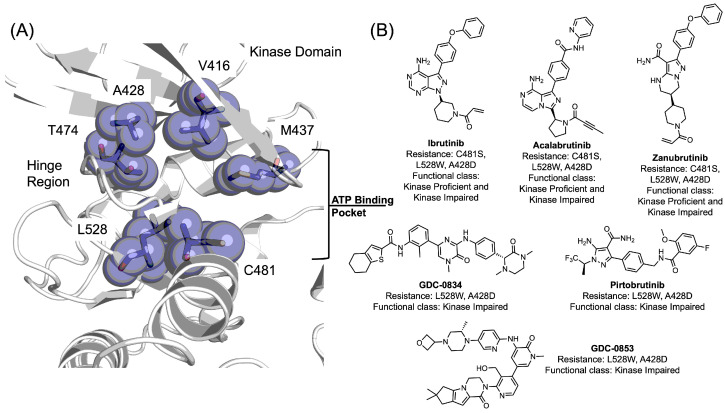
Structural location of clinically relevant BTK resistance mutations within the kinase domain and representative BTK inhibitors. (**A**) Resistance-associated mutations are mapped onto the BTK domain according to their structural location and classified as kinase-proficient or kinase-impaired, PDB:8FLL [61]. (**B**) Representative covalent and non-covalent BTK inhibitors are shown with their chemical structures [63,91,92]. This figure was made with Pymol (PyMOL. The PyMOL Molecular Graphics System, Version 2.5 Schrödinger, LLC, 2015).

A recent study published in the journal *Science* revealed that BTK possesses kinase-independent, or “kinase-dead,” functions. In these cases, the inhibition of BTK catalytic activity alone may be insufficient, as certain mutations within the kinase domain allow BTK to function as a signaling scaffold through protein–protein interactions that do not depend on its enzymatic activity. Based on these findings, BTK resistance mutations have been broadly classified into kinase-proficient mutations (C481S, T474I, Figure 4), which retain catalytic activity; and kinase-impaired mutations (L528W, V416L, A428D, M437R, Figure 4), which lose catalytic activity but maintain signaling through scaffolding functions [63,90].

**Figure 5:**
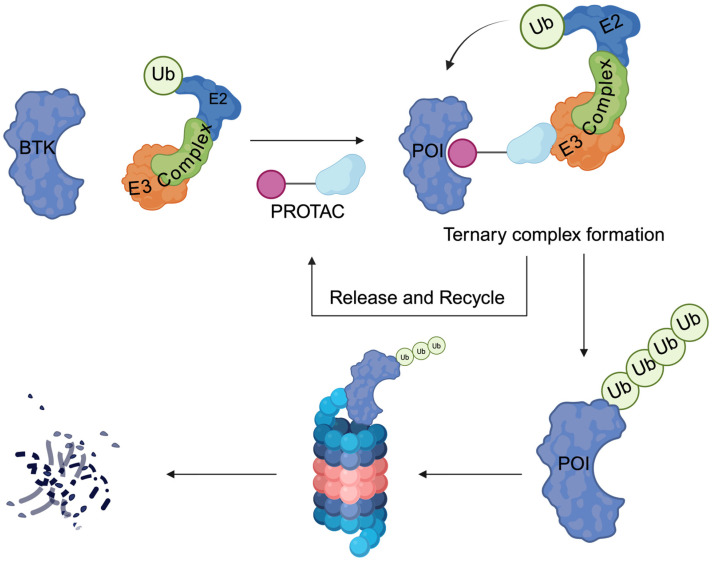
PROTAC mechanism of action. A BTK-targeting PROTAC simultaneously binds BTK and an E3 ubiquitin ligase, facilitating ternary complex formation. This interaction promotes the transfer of ubiquitin molecules onto BTK, marking it for recognition and degradation by the 26S proteasome. Unlike conventional inhibitors, PROTACs eliminate the entire BTK protein, suppressing both kinase-proficient and kinase-impaired functions [95]. Created in BioRender. Bravo Jr., E. (2026) https://BioRender.com/ojd02jw (accessed on 27 July 2026).

To address both the enzymatic and non-enzymatic functions of BTK, targeted protein degradation has emerged as an attractive therapeutic strategy (Figure 5). One notable example is the BTK degrader NX-2127, developed by Nurix Therapeutics. NX-2127 utilizes a BTK-binding warhead that engages the hinge region and induces BTK degradation, thereby eliminating both the kinase-dependent and scaffold-mediated signaling functions. Importantly, this approach demonstrated activity against a broad spectrum of clinically relevant BTK resistance mutations [71].

In parallel, several additional BTK degraders have been reported. For example, Li et al. developed a BTK PROTAC based on the GDC-0853 scaffold, which targets the H3 pocket and achieves a DC_50_ of 0.5 nM [93]. MT-802, another BTK degrader, incorporates a distinct BTK-binding scaffold that engages the back pocket and exhibits a DC_50_ of 6.2 nM [94]. These examples highlight how different binding regions within BTK can be leveraged to induce degradation while raising important questions regarding the relationship between binding site selection, kinase selectivity, ternary complex formation, and degradation efficiency. An additional advantage of targeted protein degradation is that it shifts the emphasis away from maximizing inhibitor potency. Although PROTACs still require binding to the target protein, high-affinity binding is not always necessary to achieve efficient degradation. Instead, successful degradation depends largely on the formation of a productive ternary complex between the target protein, the PROTAC, and the E3 ligase—a process that is strongly influenced by the geometry and composition of the linker. Consequently, PROTACs can retain activity against resistance-associated mutations that partially impair inhibitor binding, as even modest target engagement may be sufficient to induce ubiquitination and subsequent proteasomal degradation.

As the understanding of BTK biology continues to evolve, targeted protein degradation offers a promising strategy to overcome both catalytic and non-catalytic resistance mechanisms. Consequently, significant efforts are underway to develop next-generation BTK degraders capable of targeting wild-type BTK as well as emerging resistance-associated mutations. In addition to NX-2127, both NX-5948 [96] and BGB-16673 [97] are currently in clinical trials, highlighting the growing interest in degrading rather than simply inhibiting BTK [98]. A current challenge in BTK-targeted therapy is the emergence of the novel BTK A428D mutation, for which no clinically effective inhibitor or degrader has yet been reported. This raises the important question of how to therapeutically target this resistance mutation. Recently, Crossfire Oncology, a Netherlands-based pharmaceutical company, proposed an alternative strategy based on a macrocyclic inhibitor design. Their findings suggest that inhibition alone may be sufficient to suppress BTK signaling by inducing conformational changes within the kinase domain. These structural rearrangements may disrupt important interactions required for the non-catalytic scaffolding function of BTK, thereby preventing persistent B-cell receptor signaling even in kinase-impaired BTK mutants [91,92,99,100,101,102,103].

In parallel with these therapeutic advances, CRISPR/Cas-based genome editing technologies have emerged as powerful tools for investigating the complex biology of BTK and accelerating the development of next-generation therapeutic strategies [63,104,105]. Although clinical application remains limited by challenges related to editing specificity, off-target effects, efficient in vivo delivery and safety, CRISPR-based approaches enable precise engineering of clinically relevant resistance-associated mutations including BTK C481S, A428D, and other emerging variants identified in patients receiving covalent and non-covalent BTK inhibitors, thereby facilitating mechanistic investigations into how these alterations influence kinase activity, protein stability, downstream signaling and therapeutic response.

In addition to modeling drug resistance, CRISPR/Cas technologies provide powerful platforms for distinguishing kinase-dependent catalytic functions from the kinase independent scaffold activities of BTK. Recent evidence suggests that certain kinase-impaired BTK mutants retain the ability to promote B-cell receptor signaling through protein–protein interactions, therefore highlighting biological functions that cannot be fully suppressed by conventional inhibitors [63]. CRISPR-mediated knockout, domain-specific editing and targeted mutagenesis enable investigators to selectively disrupt catalytic domains, protein interactions, and regulatory motifs, providing insight into the structural and functional roles of BTK signaling in both normal and malignant B cells.

Overall, such functional genomic studies can identify compensatory signaling, synthetic lethal interactions, and potential biomarkers associated with response and resistance to BTK-targeted therapies. Finally, as our understanding of BTK biology continues to expand, CRISPR-based functional genomics is expected to guide the development of more durable therapeutic approaches to overcome both catalytic and non-catalytic mechanisms of resistance.

## 5. Conclusions

Bruton’s tyrosine kinase (BTK) continues to present new challenges for therapeutic intervention. From the emergence of resistance mutations that prevent effective covalent inhibition, to the difficulty of achieving selectivity among closely related kinases, and more recently the discovery of kinase-independent scaffold functions, BTK has repeatedly demonstrated its ability to evade therapeutic pressure. The identification of kinase-impaired mutations capable of sustaining signaling through non-catalytic mechanisms represents a new paradigm in BTK biology and highlights the limitations of strategies that focus solely on inhibiting kinase activity.

Given the central role of BTK in the pathogenesis of B-cell malignancies and autoimmune diseases, there remains a critical need to develop therapeutic approaches capable of overcoming both catalytic and non-catalytic resistance mechanisms. In this review, we have discussed the major challenges associated with BTK-targeted drug discovery, including irreversible inhibitor resistance, kinase selectivity, reversible inhibition strategies, and the recently identified kinase-dead scaffold functions of BTK. Together, these findings illustrate the evolving complexity of BTK biology and the need for innovative approaches to maintain durable therapeutic responses.

The evolution from first- to third-generation BTK inhibitors represents a clear trajectory of improved kinase selectivity, driven by structural modifications within the ATP-binding site, back pocket, and H3 pocket. These advances have translated directly into meaningful clinical benefits, as demonstrated by the reduced off-target toxicities observed with second-generation covalent inhibitors and the ability of non-covalent inhibitors to overcome C481S resistance. Nevertheless, the therapeutic landscape continues to be shaped by new resistance mechanisms, from non-C481 mutations (T474I, L528W) that confer cross-resistance across both covalent and non-covalent agents, to kinase-impaired mutations that sustain BCR signaling through scaffolding functions independent of catalytic activity. These findings underscore that there is no universal BTK-targeting strategy: each binding mode and selectivity profile carries distinct vulnerabilities to resistance. Moving forward, the continued development of structurally diverse inhibitors, targeted protein degraders, and emerging modalities will be essential to address the full spectrum of resistance mechanisms. Ultimately, the design of next-generation BTK therapeutics will require not only optimizing selectivity within the kinase domain but also anticipating the evolutionary pressures that drive resistance.

**Figure 6:**
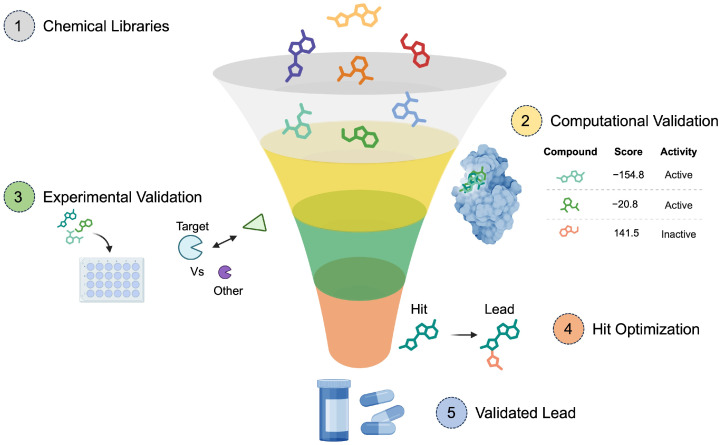
High-throughput screening pipeline for lead compound discovery. Overview of the lead discovery process combining high-throughput experimental screening technologies with computational approaches. Candidate compounds identified from large chemical libraries are experimentally validated; prioritized based on potency, selectivity, and drug-like properties; and further optimized to produce validated lead compounds for preclinical evaluation [113]. Created in BioRender. Bravo Jr., E. (2026). https://BioRender.com/ojd02jw (accessed on 27 July 2026).

Recent advances in artificial intelligence (AI) and high-throughput screening (HTS) technologies offer promising opportunities to accelerate the discovery of next-generation BTK therapeutics (Figure 6). AI-driven approaches may facilitate the prediction of resistance mutations, identify novel binding pockets, optimize inhibitor selectivity, and enable the rapid screening of compounds against both known and emerging BTK variants. High-throughput screening (HTS) approaches, including DNA-encoded libraries (DELs) and fragment-based drug discovery (FBDD) enable the rapid screening of millions to billions of compounds against a biological target, facilitating the identification of initial hits that can subsequently be prioritized based on properties such as selectivity, potency, and target occupancy [106,107,108,109,110]. These hits can then be further refined using computational approaches—such as molecular docking, scoring functions, and in silico prediction of physicochemical and pharmacokinetic properties—to prioritize compounds with favorable binding modes and drug-like characteristics. Together, the integration of AI and experimental HTS reduces the need for extensive compound synthesis and empirical screening while accelerating hit identification, lead optimization, and the development of more effective BTK inhibitors. Furthermore, a deeper understanding of the evolutionary pressures and molecular mechanisms that drive the emergence of BTK resistance mutations may reveal new strategies for preventing or delaying therapeutic escape and improve the long-term durability of BTK-targeted therapies [111,112].

Among the approaches currently under investigation, targeted protein degradation appears particularly promising. By eliminating BTK protein rather than simply inhibiting its catalytic activity, degraders have the potential to suppress both kinase-dependent and scaffold-mediated signaling functions. However, emerging mutations such as A428D remain resistant both to current BTK inhibitors and degraders, highlighting the need for alternative therapeutic strategies. Continued efforts to understand the structural and biological determinants of BTK resistance, combined with advances in computational drug discovery and induced-proximity technologies, will likely play a critical role in the development of future therapies capable of overcoming the next generation of BTK-driven disease.

## Data Availability

No new data were created or analyzed in this study. Data sharing is not applicable to this article.

## Abbreviations

The following abbreviations are used in this manuscript:

A428D	Alanine-428-to-Aspartic Acid mutation
ATP	Adenosine Triphosphate
BCR	B-cell Receptor
BLK	B-Lymphoid Kinase
BLNK	B-cell Linker
BMS-986142	Bristol-Myers Squibb-986142
BMS-935177	Bristol-Myers Squibb-935177
BMX	Bone Marrow Tyrosine Kinase on Chromosome X
BTK	Bruton’s Tyrosine Kinase
BTK WT	Bruton’s Tyrosine Kinase Wild-Type
C481	Cysteine at position 481
C481S	Cysteine-to-serine substitution at position 481
Ca^2+^	Calcium ion
CaMKII	Calcium/Calmodulin-Dependent Protein Kinase II
CD19	Cluster of Differentiation 19
CLL	Chronic Lymphocytic Leukemia
CRBN	Cereblon
CSK	C-terminal Src Kinase
D539	Aspartic Acid at position 539
DC_50_	Half-maximal degradation concentration
DELs	DNA-Encoded Libraries
D_max_	Maximum target degradation
E3	E3 ubiquitin ligase
E475	Glutamic Acid at position 475
EGFR	Epidermal Growth Factor Receptor
ERBB2	Erb-B2 Receptor Tyrosine Kinase 2
FDA	U.S. Food and Drug Administration
GDC-0834	Genentech Drug Candidate-0834
GDC-0853	Fenebrutinib
HTS	High-Throughput Screening
ITAM	Immunoreceptor Tyrosine-based Activation Motif
ITK	Interleukin-2-Inducible T-cell Kinase
JAK3	Janus Kinase 3
K430	Lysine at position 430
Lyn	Proto-oncogene, Src Family Tyrosine Kinase
M437R	Methionine-437-to-Arginine mutation
M477	Methionine at position 477
MCL	Mantle Cell Lymphoma
MZL	Marginal Zone Lymphoma
PDB	Protein Data Bank
PI3K-Akt	Phosphoinositide 3-Kinase (PI3K) and Protein Kinase B (Akt)
PI3K	Phosphatidylinositol-3 Kinase
PIP2	Phosphatidylinositol-4,5-Bisphosphate
PIP3	Phosphatidylinositol-3,4,5-Triphosphate
PLCγ2	Phospholipase C Gamma-2
POI	Protein of interest
PROTACs	PROteolysis TArgeting Chimeras
S538	Serine at position 538
SLL	Small Lymphocytic Lymphoma
Src/SRC	Family of Proto-oncogene Non-receptor Tyrosine Kinases
Syk	Spleen Tyrosine Kinase
T474	Threonine at position 474
T474I	Threonine-474-to-Isoleucine mutation
TEC	Tyrosine Kinase Expressed in Hepatocellular Carcinoma
TXK	Tyrosine-Protein Kinase
Tyr223	Tyrosine at position 223
Tyr551	Tyrosine at position 551
V416L	Valine-416-to-Leucine mutation
VHL	von Hippel–Lindau E3 ubiquitin ligase
WM	Waldenström Macroglobulinemia
